# *Limosilactobacillus fermentum* MG5368 and *Lactiplantibacillus plantarum* MG989 Regulates Skin Health in UVB-Induced HaCaT Cells and Hairless Mice Model

**DOI:** 10.3390/nu16234083

**Published:** 2024-11-27

**Authors:** Jeong-Yong Park, Ji Yeon Lee, Seonghwa Hong, Huijin Heo, Hana Lee, Yong Gyeong Kim, Byoung-Kook Kim, Soo-Im Choi, Junsoo Lee

**Affiliations:** 1 Mediogen, Co., Ltd., Biovalley 1-ro, Jecheon-si 27159, Republic of Korea; pjy@mediogen.co.kr (J.-Y.P.); ljy@mediogen.co.kr (J.Y.L.); kyk@mediogen.co.kr (Y.G.K.); kbk@mediogen.co.kr (B.-K.K.); 2 Department of Food Science and Biotechnology, Chungbuk National University, Cheongju-si 28644, Republic of Korea; tjd3465@naver.com (S.H.); pltreasure11@gmail.com (H.H.); dlgksk0514@naver.com (H.L.)

**Keywords:** ultraviolet B, wrinkle, collagen, *Limosilactobacillus fermentum*, *Lactiplantibacillus plantarum*

## Abstract

Background: Photoaging, induced by chronic ultraviolet B (UVB) exposure, results in the degradation of extracellular matrix (ECM) components, leading to skin roughness, wrinkle formation, and reduced elasticity. Recent studies have explored probiotics as potential inhibitors of extrinsic aging, primarily through mechanisms that protect the skin barrier and reduce collagen breakdown. Methods: This study investigates the anti-photoaging effects of *Limosilactobacillus fermentum* MG5368 (*L. fermentum* MG5368) and *Lactiplantibacillus plantarum* MG989 (*L. plantarum* MG989) in UVB-exposed keratinocytes and an SKH-1 hairless mice model. Results: Both strains demonstrated significant efficacy in preserving collagen through the inhibition of activating protein-1 (AP-1) and reducing the expression of matrix metalloproteinase (MMP)-1 and MMP-3. Additionally, both strains restored COL1A1 protein expressions, thereby enhancing collagen synthesis and ECM stability. Enhanced skin elasticity was observed, attributed to restored levels of hyaluronic acid and hyaluronan synthase 2 (HAS2) protein expressions. Conclusions: These findings suggest that *L. fermentum* MG5368 and *L. plantarum* MG989 may serve as promising probiotic-based agents for anti-photoaging applications.

## 1. Introduction

As the largest organ of the human body, the skin constitutes approximately 16% of total body mass and functions as the first line of defense against external environmental factors [1]. The skin consists of two main layers, the epidermis and the dermis, with the epidermis being the outermost layer and acting as a barrier against environmental stressors such as ultraviolet (UV) exposure [2]. Epidermal keratinocytes migrate outward to form keratinocytes, forming the main barrier of the epidermal layer [3]. Skin aging involves a progressive decline in both structural integrity and physiological function, generally categorized into intrinsic aging, associated with natural biological processes, and extrinsic aging, driven by environmental factors such as pollution and UV exposure [4]. Among the types of UV radiation, UVB has particularly pronounced effects on skin health, contributing to dryness, hyperpigmentation, and wrinkle formation through its rapid interaction with cellular processes [5]. The most characteristic change in aged skin is a decline in both the quantity and structural alteration of collagen, primarily due to accelerated degradation processes and reduced biosynthesis [6]. Recently, some natural extracts have been used as dietary ingredients to prevent photoaging, but they have been reported to have high prices and side effects. [7]. Therefore, there is considerable interest in identifying cost-effective and safe materials to mitigate skin damage and photoaging caused by UV exposure.

In general, probiotics are beneficial bacteria that eliminate harmful microorganisms and benefit the health of the host [8]. Probiotics maintain the natural balance of gut bacteria and modulate immune responses [9]. Probiotics maintain the natural balance of gut bacteria and modulate immune responses [10]. Probiotics have been extensively studied for their gut health benefits, but recent studies have also shown that they have the potential to address antioxidant, anti-inflammatory, and immune properties [11,12]. In particular, probiotics such as *Limosilactobacillus fermentum* (*L. fermentum*) and *Lactiplantibacillus plantarum* (*L. plantarum*) have been reported to have high antioxidant potential [13]. Substances with antioxidant properties have a high potential for improving skin health caused by photoaging [14]. Therefore, this study investigated the protective effects and potential mechanisms of *L. fermentum* and *L. plantarum* strains against UVB-exposed keratinocytes and SKH-1 hairless mice.

## 2. Materials and Methods

### 2.1. Preparation of Bacterial Samples

The eight bacterial strains (*L. fermentum* MG5368, MG4294, MG5159, MG4538, MG5091, *L. plantarum* MG989, MG5530, and MG5023) were isolated from healthy infant feces or breast milk (Table 1). These strains were inoculated in Man Rogosa Sharpe broth (Difco, Sparks, MD, USA) and cultured at 37 °C for 18 h. The strains were centrifuged at 1500× *g* for 10 min, and the cell-free supernatant (CFS) was collected and filtered with a 0.22 μm polytetrafluoroethylene membrane filter (ADVANTEC, Tokyo, Japan). The prepared CFS was used for the in vitro study [15]. For the in vivo test, the harvested *L. fermentum* MG5368 and *L. plantarum* MG989 pellets were freeze-dried. The powdered strains were harvested, mixed with maltodextrin for dilution at a final concentration of 1 × 10^11^ CFU/g, and stored at 4 °C until further use.

### 2.2. In Vitro Test

#### 2.2.1. Cell Culture

The HaCaT keratinocytes (provided by the School of Cosmetic Science and Beauty Biotechnology, Semyung University, Jecheon, Republic of Korea) and HT-29 cells (Korea Cell Line Bank, Seoul, Republic of Korea) were cultured as described in previous reports [16].

#### 2.2.2. Ultraviolet B (UVB) Exposure and Cell Viability

The HaCaT keratinocytes were seeded in a 96-well plate (1 × 10^4^ cell/well) and incubated at 37 °C for 24 h. Then, CFS of probiotics samples, 3% in serum-free DMEM (*v*/*v*), were pre-treated for 24 h. The cells were then irradiated with UVB (20 mJ/cm^2^) using a UVB lamp (Sankyo Denki Lamps, GL20SE, Marine, Kanagawa, Japan). The intensity was monitored using a UV light meter (LT Lutron, UV-340A, Taipei, Taiwan). After exposure, cells were treated with probiotics samples for an additional 24 h [17]. Following treatment, 3-(4,5-dimethylthiazol-2-yl)-2,5-diphenyltetrazolium bromide (MTT) was added to each well, and cells were incubated for 2 h [18]. After incubation, the formazan was dissolved in dimethyl sulfoxide. The absorbance was measured at 550 nm using a microplate reader (BioTek, Winooski, VT, USA).

### 2.3. In Vivo Test

#### 2.3.1. Animal Experiment and UVB Exposure

**Figure 1:**
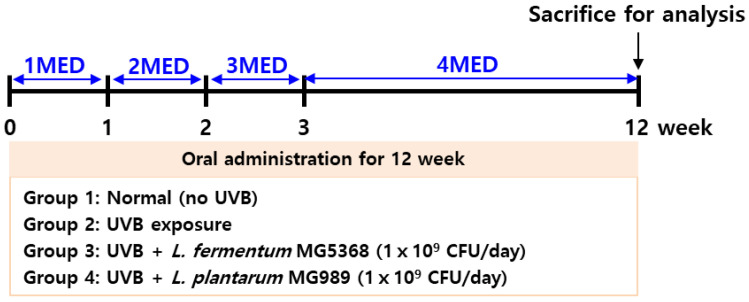
Animal experimental procedure of UVB-exposed SKH-1 hairless mice. The dorsal skin of the SKH-1 mice was exposed to UVB at 1 minimal erythema dose (MED) was 60 mJ/cm^2^.

The 5-week-old SKH-1 female hairless mice (OrientBio Co., Ltd., Seongnam, Republic of Korea) were housed in the following environment (22 ± 2 °C, 50 ± 10% humidity, and less than 60 dB with a 12 h photoperiod and free access to food and water). The experimental animals were divided into four groups so that the average body weight and standard deviation of each group were uniform (*n* = 8): (1) normal group (no UVB exposure), (2) UVB exposure group, (3) i.p. *L. fermentum* MG5368 (1 × 10^9^ CFU/day) with UVB exposure group, and (4) i.p. *L. plantarum* MG989 (1 × 10^9^ CFU/day) with UVB exposure group. Each probiotic strain was orally administered for 12 weeks with UVB exposure. The animals were exposed to UVB 3 times per week for 12 weeks, and total irradiation was 7.56 J/cm^2^ using a UV Crosslinking Chamber (UVP CL-1000, Upland, CA, USA). The UVB irradiation started with 1 Minimal Erythema Dose (MED; 0.06 J/cm^2^) during the first week and increased by 1 MED every week until the 4 weeks. UVB exposure was maintained at 4 MED from 4 to 12 weeks shown in Figure 1. [19]. The UVB intensity was monitored by a UV light meter (VLX-3W radiometer, UVITEC, Milton, Cambridge, UK). There were no deaths by the end of the experiment. At the end of the animal experiment, all mice were anesthetized with 3% isoflurane, and skin wrinkle and elasticity were assessed with no exclusions.

#### 2.3.2. Evaluation of Skin Wrinkle and Skin Elasticity

The dorsal skin exposed to UVB was imaged using a 3D imaging system equipped with a high-resolution sensor (Primos^CR^, Canfield, OH, USA) [20]. The mean of wrinkle depth, maximum of wrinkle depth, and skin roughness (Rz) were analyzed by Primos 5.0 software (Canfield, OH, USA). The dorsal skin elasticity was measured by a Cutometer (Courage and Khazaka, Kőln, Germany) [21]. The measuring mode was used with a constant negative pressure of 450 mbar consecutively three times of suction for two seconds each. The parameters of R2 (gross elasticity), R5 (net elasticity), and R7 (biological elasticity) were analyzed.

#### 2.3.3. Analysis of Histopathology and Collagen Content

The dorsal skin was biopsied, fixed with 10% paraformaldehyde, and embedded in paraffin block. The slides were made by sectioning into 3 μm thick and subjected to a hydration process. The slides of skin dorsal tissues were stained with hematoxylin and eosin (H&E) solution to measure skin thickness. The collagen production was stained with Biebrich Scarlet-acid Fuchsin solution for 5 min, washed, and stained with phosphotungtic/phosphomolybdic acid for 5 min [22]. All stained tissue was pictured using an optical microscope (Olympus, Tokyo, Japan). The thickness of the skin and the production of collagen were analyzed by the ImageJ program (https://imagej.net/ij/, The National Institutes of Health, Bethesda, MD, USA).

### 2.4. Enzyme-Linked Immunosorbent Assay (ELISA)

In keratinocytes, matrix metalloproteinase (MMP)-1, MMP-3 (Merck & Co. Inc., Whitehouse Station, NJ, USA), and collagen content (Sircol Soluble Collagen Assay Kit, Biocolor, Belfast, UK) production were measured by according to the manufacturer’s instructions. Hyaluronic acid (HA) contents in keratinocytes and dorsal skin were evaluated by using an HA ELISA assay kit (R&D systems, Minneapolis, MN, USA) according to the manufacturer’s instructions.

### 2.5. Western Blotting

Western blot antibodies were obtained from Cell Signaling Technology (Beverly, MA, USA) and Santa Cruz Biotechnology (Dallas, TX, USA). The HaCaT keratinocytes and dorsal skin were lysed with a Pro-Prep protein extraction solution (iNtRON, Seongnam-si, Republic of Korea). Proteins were separated using SDS-PAGE and transferred onto an NC membrane (Cytiva, Marlborough, MA, USA). The membranes were blocked with 5% bovine serum albumin (BSA; fatty acid-free, purity ≥98%) for 1 h at room temperature and subsequently incubated 24 h at 4 °C with primary antibodies at dilutions of 1:500. After primary antibody incubation, the blots were incubated with secondary antibodies diluted 1:1000 for 1 h. Chemiluminescence was detected using ECLTM (ThermoFisher, Waltham, MA, USA). Specific protein bands were quantified using ATTO CS Analyzer 4 software (ATTO, Tokyo, Japan).

### 2.6. Statistical Analysis

GraphPad Prism 10 (GraphPad Software Inc., Boston, MA, USA) was used for all statistical analyses. All data are reported as the mean ± standard error of mean (SEM) for at least three replicates. Parametric data were analyzed by one-way analysis of variance (ANOVA) followed by Dunnett’s multiple comparison test.

## 3. Results

### 3.1. Cell Protective Effects of CFS from Bacterial Strains in UVB-Exposed HaCaT Keratinocytes Including MMPs, HA, and Collagen Production

**Figure 2:**
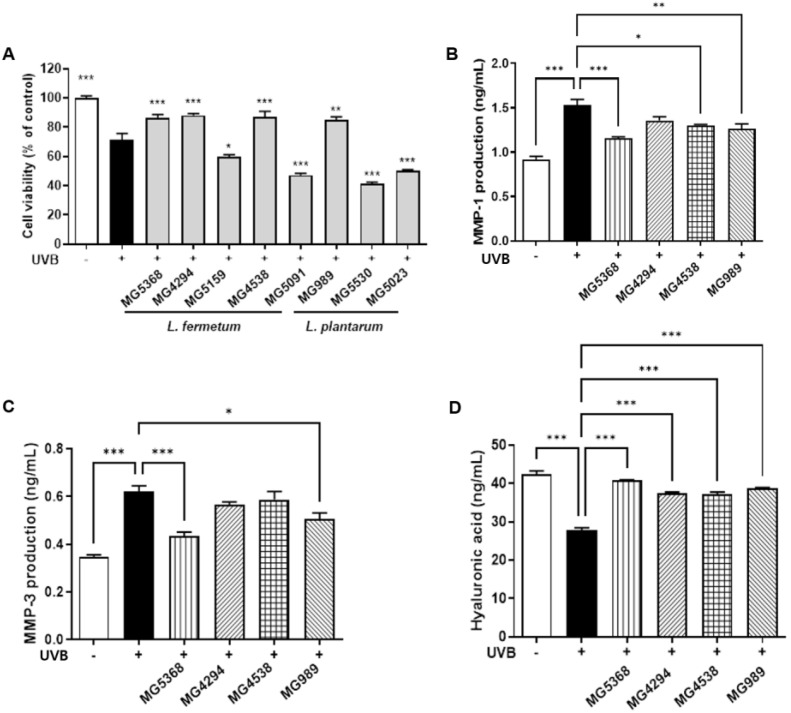
Effect on (**A**) cell viability, (**B**) MMP-3, (**C**) MMP-1, and (**D**) hyaluronic acid production of CFS in UVB-exposed HaCaT keratinocytes. The cells were pre-treated with CFS (3%) for 24 h and then exposed to UVB (20 mJ/cm^2^), followed by additional treatment with CFS (3%) for 24 h. Values are expressed as the mean ± SEM (*n* = 3). * *p <* 0.05, ** *p <* 0.01, and *** *p <* 0.001, significant difference compared to UVB-exposed group. White bar, no-exposed group; black bar, UVB-exposed group.

To determine the appropriate concentration of CFS, cell viability was assessed without UVB irradiation. To establish the UVB intensity, cell viability was measured following exposure to 10, 20, and 30 mJ/cm^2^. With a cell viability of 74.7% observed at 20 mJ/cm^2^, this intensity was chosen for subsequent experiments (Figure S1). The protective effect of CFS on UVB-exposed HaCaT keratinocytes was evaluated, and the results showed that CFS of *L. fermentum* MG5368, MG4294, and MG4538 and *L. plantarum* MG989 did not exhibit a significant recovery in cell viability (Figure 2A). The evaluation of MMP-1 and MMP-3 production in the four strains revealed that CFS of *L. fermentum* MG5368 and *L. plantarum* MG989 were significantly regulated in UVB-exposed HaCaT keratinocytes (Figure 2B,C). Additionally, *L. fermentum* MG5368 and *L. plantarum* MG989 significantly restored the collagen production levels that were reduced by UVB irradiation in HaCaT keratinocytes (Figure S2). The HA production was significantly increased in UVB-exposed HaCaT keratinocytes treated with CFS of the four strains (Figure 2D).

### 3.2. CFS of L. fermentum MG5368 and L. plantarum MG989 Regulates the AP-1/Smad Signaling Pathway in UVB-Exposed HaCaT Keratinocytes

**Figure 3:**
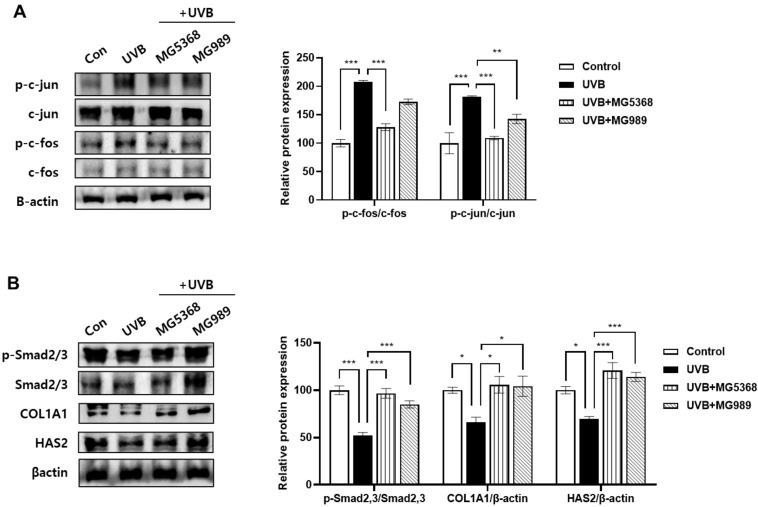
Effect of CFS on (**A**) AP-1 (p-c-fos, c-fos, p-c-jun, and c-jun) and (**B**) p-Smad 2/3, Smad 2/3, COL1A1, and HAS2 in UVB-exposed HaCaT keratinocytes. The cells were pre-treated with CFS (3%) for 24 h and then exposed to UVB (20 mJ/cm^2^), followed by additional treatment with CFS (3%) for 24 h. The representative blotting images and relative expression were evaluated by Western blotting. The β-actin was used as a loading control. Values are expressed as the mean ± SEM (*n* = 3). * *p <* 0.05, ** *p <* 0.01, and *** *p <* 0.001, significant difference compared to the UVB-exposed group.

In HaCaT keratinocytes, the activator protein (AP)-1 signaling pathway, including phosphorylation of c-Fos and c-Jun protein expression, stimulated by UVB exposure was significantly reversed by CFS of *L. fermentum* MG5368 and *L. plantarum* MG989 (Figure 3A). CFS of *L. fermentum* MG5368 and *L. plantarum* MG989 also strongly increased phosphorylation of Smad 2/3, collagen type I, alpha 1 (COL1A1), and hyaluronan synthase (HAS) 2 protein expression in UVB-exposed HaCaT keratinocytes (Figure 3B).

### 3.3. Oral Administration of L. fermentum MG5368 and L. plantarum MG989 Decreases Wrinkle and Elasticity in the Dorsal Skin of the UVB-Exposed SKH-1 Hairless Mice

**Figure 4:**
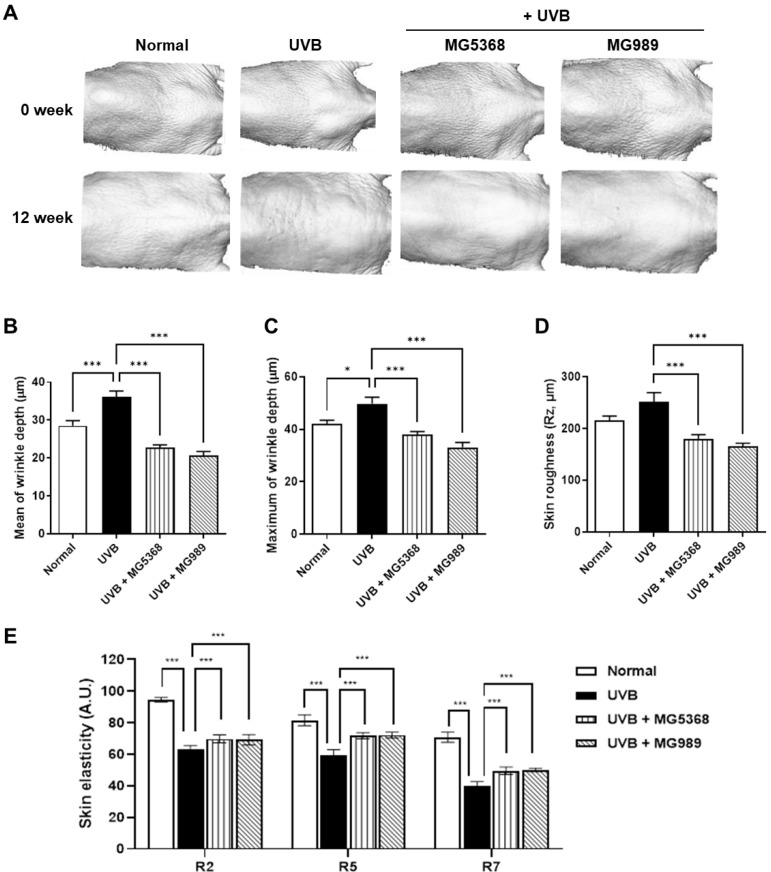
Effect of *L. fermentum* MG5368 and *L. plantarum* MG989 on wrinkle formation, roughness, and elasticity in UVB-exposed SKH-1 hairless mice. HR-1 hairless mice were orally administrated with *L. fermentum* MG5368 and *L. plantarum* MG989 (1 × 10^9^ CFU/day) and were irradiated with UVB for 12 weeks. (**A**) Representative photographs of the mouse dorsal skin. (**B**) Mean of wrinkle depth, (**C**) maximum of wrinkle depth, (**D**) skin roughness (Rz), and (**E**) skin elasticity (A.U.) were analyzed. Values are expressed as the mean ± SEM (*n* = 8). * *p <* 0.05, and *** *p <* 0.001, significant difference compared to UVB-exposed group.

Wrinkle formation was similar prior to UVB exposure; however, after 12 weeks of UVB exposure, wrinkles developed in the dorsal skin of SKH-1 hairless mice. Oral administration of *L. fermentum* MG5368 and *L. plantarum* MG989 significantly reduced wrinkle formation induced by UVB exposure at 12 weeks (Figure 4A). The mean and maximum wrinkle depth and skin roughness in UVB-exposed SKH-1 hairless mice were improved by the intake of *L. fermentum* MG5368 and *L. plantarum* MG989 (Figure 4B–D). The R2, R5, and R7, which indicate skin elasticity parameters, showed a decrease following UVB exposure; however, oral administration of *L. fermentum* MG5368 and *L. plantarum* MG989 significantly increased these parameters (Figure 4E).

### 3.4. Oral Administration of L. fermentum MG5368 and L. plantarum MG989 Reduces Epidermis Thickness, Collagen, and HA in the Dorsal Skin of the UVB-Exposed SKH-1 Hairless Mice

**Figure 5:**
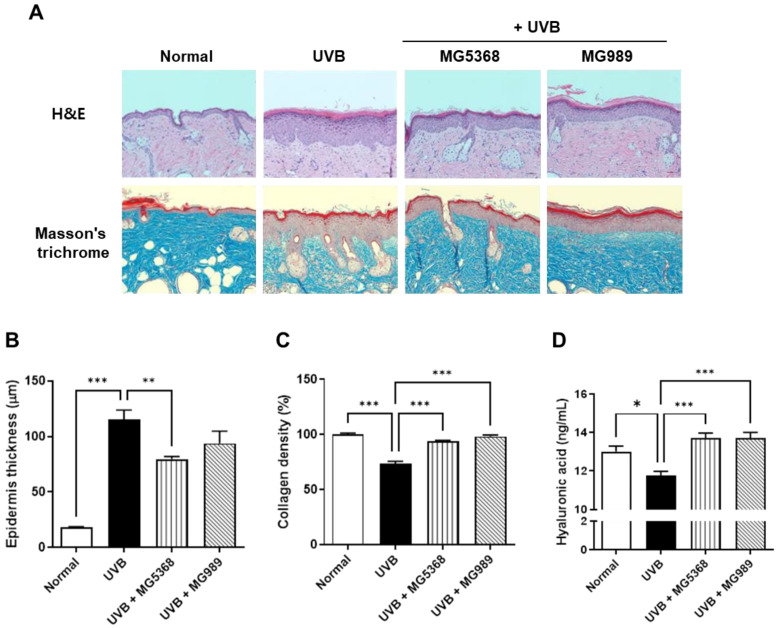
Histological observation and hyaluronic acid (HA) contents on SKH-1 hairless mice dorsal skin orally administrated with *L. fermentum* MG5368 and *L. plantarum* MG989 (1 × 10^9^ CFU/day) and were exposed with UVB for 12 weeks. (**A**) Representative images of H&E and Masson’s trichrome (MT) staining of the mouse dorsal skin. (**B**) Epidermis thickness by H&E stain, (**C**) collagen density by Masson’s trichrome stain, and (**D**) HA analyzed by ELISA. Values are expressed as the mean ± SEM (*n* = 8). * *p <* 0.05, ** *p <* 0.01, and *** *p <* 0.001, significant difference compared to UVB-exposed group.

Histological changes visualized by H&E and MT staining and HA levels in dorsal skin are shown in Figure 5. After UVB exposure to the dorsal skin of SKH-1 hairless mice, epidermis thickness was significantly increased, while oral administration of *L. fermentum* MG5368 significantly reduced epidermis thickness via H&E staining. MT staining revealed collagen fiber deposition and organization, which were quantified to measure collagen density. The collagen density was reduced by UVB exposure; however, the administration of *L. fermentum* MG5368 and *L. plantarum* MG989 significantly restored these levels to those observed in the normal group. Additionally, the administration of *L. fermentum* MG5368 and *L.s plantarum* MG989 significantly increased HA levels that had been reduced by UVB exposure.

### 3.5. Oral Administration of L. fermentum MG5368 and L. plantarum MG989 Regulated AP-1/Smad Signaling Pathway in the Dorsal Skin of the UVB-Exposed SKH-1 Hairless Mice

**Figure 6:**
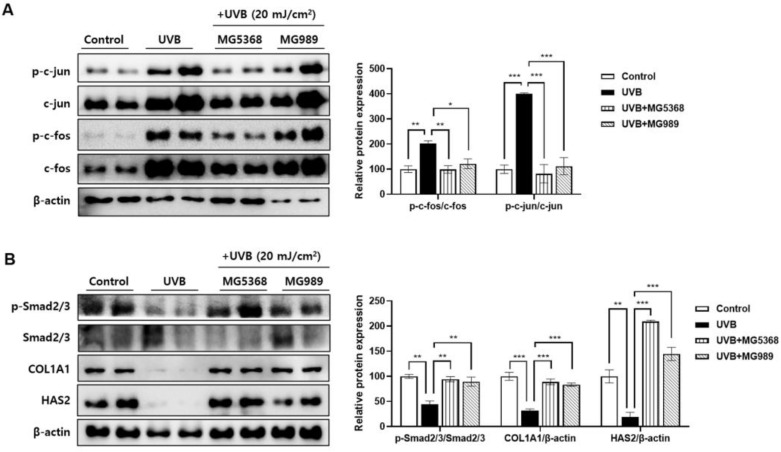
Effects of *L. fermentum* MG5368 and *L. plantarum* MG989 on the AP-1/Smad pathway in UVB-exposed SKH-1 hairless mice. HR-1 hairless mice were orally administrated with *L. fermentum* MG5368 and *L. plantarum* MG989 (1 × 10^9^ CFU/day) and were irradiated with UVB for 12 weeks. The representative blotting images and relative expression of (**A**) AP-1 (p-c-fos, c-fos, p-c-jun, and c-jun) and (**B**) p-Smad 2/3, Smad 2/3, COL1A1, and HAS2 were evaluated by Western blotting. The β-actin was used as a loading control. Values are expressed as the mean ± SEM (*n* = 8). * *p <* 0.05, ** *p <* 0.01, and *** *p <* 0.001, significant difference compared to UVB-exposed group.

In UVB-exposed dorsal skin of mice, phosphorylation of c-fos and c-jun, including in the AP-1 signaling pathway, was significantly reduced by the administration of *L. fermentum* MG5368 and *L. plantarum* MG989 (Figure 6A). Additionally, administration of *L. fermentum* MG5368 and *L. plantarum* MG989 significantly increased the phosphorylation of Smad 2/3 and the expression of COL1A1 and HAS2 proteins, which were reduced by UVB exposure (Figure 6B).

## 4. Discussion

One of the strategies to prevent photoaging is to use natural substances that can inhibit collagen breakdown. Recently, probiotics, rather than synthetic substances, have been studied extensively as alternatives with skin-preventing effects against extrinsic aging [1]. The *Lactobacillus* genus is commonly found and may be beneficial to the skin [23]. Probiotics attached to the gut can influence the microbial community, and probiotic metabolites, including lipoteichoic acid, lactic acid, and acetic acid, improve the skin barrier [8]. *L. plantarum* HY7714 maintained skin moisture and prevented wrinkles in vitro, in vivo, and in clinical trials [24,25]. Our previous study demonstrated that *Lactobacillus fermentum* MG5368 exhibited photoprotective effects against UVB exposure in human dermal fibroblasts, while *Lactobacillus plantarum* MG989 showed significant radical-scavenging activity and anti-inflammatory effects in RAW264.7 cells stimulated with LPS [8,26]. Nevertheless, the molecular mechanisms of photoprotective effects from *L. fermentum* MG5368 and *L. plantarum* MG989 remain unclear. Therefore, in this study, we investigated the photoprotective mechanism of *L. fermentum* MG5368 and *L. plantarum* MG989 in keratinocytes and in vivo.

One of the mechanisms for preventing skin aging is to maintain the skin barrier components [7]. Recent studies have shown that extracellular matrix (ECM) degradation is a major factor in wrinkle formation, specifically inducing ECM structural changes and dehydration [27]. Chronic UVB exposure induces collagen degradation, which leads to skin roughness, wrinkles, and loss of elasticity [28,29]. *L. fermentum* MG5368 and *L. plantarum* MG989 significantly restored collagen in keratinocytes and in vivo models against UVB. In addition, *L. fermentum* MG5368 and *L. plantarum* MG989 restored the average and maximum wrinkle depth, skin roughness, and elasticity parameters R2, R5, and R7 to normal levels. The thickening of the epidermis and dermis is a primary defensive response to UV exposure, with prolonged UV exposure leading to an increase in the thickness of both the epidermal and dermal layers [30]. In our study, *L. fermentum* MG5368 and *L. plantarum* MG989 restored epidermis thickness in a UVB-exposed in vivo model. These findings indicate that the two strains improved wrinkle-related parameters by enhancing collagen production, a key component of the skin barrier.

Complex signaling induced by UV exposure leads to an increase in the expression of MMPs, which in turn promotes the degradation of proteins that constitute the ECM [31]. In the various types of MMP enzymes, UV exposure increases the expression of MMP-1 and MMP-3 in the dermal fibroblast and keratinocytes [32,33]. MMP-1 is a key enzyme in the collagen degradation process, cleaving collagen into two fragments and initiating collagen breakdown [34]. MMP-3, also known as stromelysin-1, degrades various types of ECM and activates MMP enzymes [33]. AP-1, composed of subunits of c-fos and c-jun, is activated by UVB and acts as a transcription factor that activates MMPs [7]. UVB exposure also damages the Smad 2/3 signaling pathway, resulting in decreased collagen synthesis [30]. Phosphorylation of Smad 2 and Smad 3 prompts their translocation from the cytoplasm to the nucleus and induces gene expression for collagen and collagen receptors, including COL1A1, thereby regulating collagen synthesis [35,36]. In our study, we investigated the effects on collagen production and degradation. *L. fermentum* MG5368 and *L. plantarum* MG989 were identified to have a molecular mechanism to reduce MMP-1 and MMP-3 by inhibiting AP-1 in UVB-exposed keratinocytes. In addition, these strains were identified to upregulate COL1A1 in UVB-exposed keratinocytes and in vivo models, thereby regulating the expression of factors involved in both collagen production and degradation.

An additional approach to prevent skin aging involves enhancing factors that maintain skin hydration levels [7]. HA is a crucial component of the ECM, essential for maintaining skin hydration and elasticity [37]. Exposure to UVB accelerates the breakdown of HA, leading to decreased moisture levels and reduced elasticity in the skin [38]. HAS2 is one of the primary enzymes that induces HA production, and it has a major role in epidermal cells [39]. In our study, *L. fermentum* MG5368 and *L. plantarum* MG989 significantly restored HA expression by upregulating HAS2 in UVB-exposed epidermal keratinocytes, with levels returning to normal in the in vivo models.

In this study, we confirmed the protective and anti-aging effects of *L. fermentum* MG5368 and *L. plantarum* MG989 against UVB-exposed skin damage. These strains contributed to collagen maintenance by inhibiting the expression of MMP-1 and MMP-3 and reducing collagen degradation by inhibiting the AP-1 pathway while promoting COL1A1 expression to maintain the ECM component and strengthen the skin barrier. Additionally, these strains positively affected skin elasticity by influencing skin moisture by restoring HA and HAS2 expression. Therefore, this study suggests that *L. fermentum* MG5368 and *L. plantarum* MG989 have the potential to be utilized as probiotic-based skin aging prevention agents, and further clinical trials and safety are needed to verify. However, further clinical trials are needed to validate the efficacy and safety of these strains.

## 5. Conclusions

In conclusion, this study demonstrates the potential of *L. fermentum* MG5368 and *L. plantarum* MG989 as effective probiotics for preventing UVB-induced skin aging. These strains exhibit significant protective effects on skin health by promoting collagen preservation and reducing ECM degradation. Specifically, *L. fermentum* MG5368 and *L. plantarum* MG989 effectively inhibited MMP-1 and MMP-3 expression by downregulating AP-1 activation, thereby mitigating collagen breakdown in UVB-exposed keratinocytes and in vivo models. Furthermore, these strains enhanced COL1A1 expression by regulating the Smad 2/3 pathway, promoting collagen synthesis and maintaining skin elasticity while inhibiting wrinkle formation. Additionally, they restored HA and HAS2 levels, which are essential for skin hydration and further supporting elasticity. These findings suggest that *L. fermentum* MG5368 and *L. plantarum* MG989 hold promise as effective alternatives for skin aging prevention.

## Figures and Tables

**Table 1:** The origin and accession number of bacterial strains used in this study.

Spp.	Strain	Origin	NCBI Accession No.
*Limosilactobacillus fermentum*	MG5368	Food	ON631268.1
	MG4294	Human	MW404502.1
	MG5159	Food	MN435579.1
	MG4538	Human	MN368558.1
	MG5091	Food	OP102518.1
*Lactiplantibacillus plantarum*	MG989	Human	MN061270.1
	MG5530	Food	ON668221.1
	MG5023	Food	OP102478.1

## Data Availability

The datasets generated during and/or analyzed during the current study are available from the corresponding author upon reasonable request.

## Supplementary Materials

The following supporting information can be downloaded at https://www.mdpi.com/article/10.3390/nu16234083/s1, Figure S1: The cell viability according to UVB exposure in HaCaT keratinocytes. Figure S2: Effect on collagen content of CFS in UVB-exposed HaCaT keratinocytes.

## References

[1] Lee J.Y., Park J.-Y., Kim Y., Kang C.-H. (2022). Protective effect of Bifidobacterium animalis subs. lactis MG741 as probiotics against UVB-exposed fibroblasts and hairless mice. Microorganisms.

[2] D’Orazio J., Jarrett S., Amaro-Ortiz A., Scott T. (2013). UV radiation and the skin. Int. J. Mol. Sci..

[3] Proksch E., Brandner J.M., Jensen J.M. (2008). The skin: An indispensable barrier. Exp. Dermatol..

[4] Park J.-Y., Lee J.Y., Kim Y., Kang C.-H. (2023). *Latilactobacillus sakei* Wikim0066 protects skin through MMP regulation on UVB-irradiated in vitro and in vivo model. Nutrients.

[5] Ichihashi M., Ando H., Yoshida M., Niki Y., Matsui M. (2009). Photoaging of the skin. Anti-Aging Med..

[6] Quan T., Fisher G.J. (2015). Role of age-associated alterations of the dermal extracellular matrix microenvironment in human skin aging: A mini-review. Gerontology.

[7] Han H.-S., Shin J.-S., Myung D.-B., Ahn H.S., Lee S.H., Kim H.J., Lee K.-T. (2019). *Hydrangea serrata* (Thunb.) Ser. extract attenuate UVB-induced photoaging through MAPK/AP-1 inactivation in human skin fibroblasts and hairless mice. Nutrients.

[8] Park J.-Y., Lee J.Y., Kim Y., Kang C.-H. (2022). Lactic acid bacteria improve the photoprotective effect via MAPK/AP-1/MMP signaling pathway on skin fibroblasts. Microorganisms.

[9] Yu Y., Dunaway S., Champer J., Kim J., Alikhan A. (2020). Changing our microbiome: Probiotics in dermatology. Br. J. Dermatol..

[10] Jwo J.Y., Chang Y.T., Huang Y.C. (2023). Effects of probiotics supplementation on skin photoaging and skin barrier function: A systematic review and meta-analysis. Photodermatol. Photoimmunol. Photomed..

[11] Morshedi M., Hashemi R., Moazzen S., Sahebkar A., Hosseinifard E.-S. (2019). Immunomodulatory and anti-inflammatory effects of probiotics in multiple sclerosis: A systematic review. J. Neuroinflammation.

[12] Kim K.-T., Kim J.-W., Kim S.-I., Kim S., Nguyen T.H., Kang C.-H. (2021). Antioxidant and anti-inflammatory effect and probiotic properties of lactic acid bacteria isolated from canine and feline feces. Microorganisms.

[13] Mishra V., Shah C., Mokashe N., Chavan R., Yadav H., Prajapati J. (2015). Probiotics as potential antioxidants: A systematic review. J. Agric. Food Chem..

[14] Calvo M.J., Navarro C., Durán P., Galan-Freyle N.J., Parra Hernández L.A., Pacheco-Londoño L.C., Castelanich D., Bermúdez V., Chacin M. (2024). Antioxidants in Photoaging: From Molecular Insights to Clinical Applications. Int. J. Mol. Sci..

[15] Kim S., Lee J.Y., Jeong Y., Kang C.-H. (2022). Antioxidant activity and probiotic properties of lactic acid bacteria. Fermentation.

[16] Lee J.Y., Park Y., Jeong Y., Kang H. (2023). Anti-Inflammatory Response in TNFα/IFNγ-Induced HaCaT Keratinocytes and Probiotic Properties of *Lacticaseibacillus rhamnosus* MG4644, *Lacticaseibacillus paracasei* MG4693, and *Lactococcus lactis* MG5474. J. Microbiol. Biotechnol..

[17] Xu X., Ding Z., Pu C., Kong C., Chen S., Lu W., Zhang J. (2024). The structural characterization and UV-protective properties of an exopolysaccharide from a Paenibacillus isolate. Front. Pharmacol..

[18] Lee J.Y., Kim Y., Kim J.-I., Lee H.-Y., Moon G.-S., Kang C.-H. (2022). Improvements in human keratinocytes and antimicrobial effect mediated by cell-free supernatants derived from probiotics. Fermentation.

[19] Kim J.-O., An G., Choi J.-H. (2024). Protective effect of mixture of *Acanthopanax sessiliflorum* and *Chaenomeles sinensis* against ultraviolet B-induced photodamage in human fibroblast and hairless mouse. Food Sci. Biotechnol..

[20] Carlos da Silva G., Barbosa M.B., Júnior F.B.C., Moreira P.L., Werka R., Martin A.A. (2023). Detection of skin wrinkles and quantification of roughness using a novel image processing technique from a dermatoscope device. Ski. Res. Technol..

[21] Kim Y.J., Lee J.O., Kim S.Y., Lee J.M., Lee E., Na J., Yoo K.H., Park S.J., Kim B.J. (2023). Effect of A. polygama APEE (*Actinidia polygama* ethanol extract) or APWE (*Actinidia polygama* water extract) on wrinkle formation in UVB-irradiated hairless mice. J. Cosmet. Dermatol..

[22] Arnal-Forné M., Molina-García T., Ortega M., Marcos-Garcés V., Molina P., Ferrández-Izquierdo A., Sepulveda P., Bodí V., Ríos-Navarro C., Ruiz-Saurí A. (2024). Changes in human skin composition due to intrinsic aging: A histologic and morphometric study. Histochem. Cell Biol..

[23] Lolou V., Panayiotidis M.I. (2019). Functional role of probiotics and prebiotics on skin health and disease. Fermentation.

[24] Ra J., Lee D.E., Kim S.H., Jeong J.-W., Ku H.K., Kim T.-Y., Choi I.-D., Jeung W., Sim J.-H., Ahn Y.-T. (2014). Effect of oral administration of *Lactobacillus plantarum* HY7714 on epidermal hydration in ultraviolet B-irradiated hairless mice. J. Microbiol. Biotechnol..

[25] Lee D.E., Huh C.-S., Ra J., Choi I.-D., Jeong J.-W., Kim S.-H., Ryu J.H., Seo Y.K., Koh J.S., Lee J.-H. (2015). Clinical evidence of effects of *Lactobacillus plantarum* HY7714 on skin aging: A randomized, double blind, placebo-controlled study. J. Microbiol. Biotechnol..

[26] Kang C.-H., Kim J.-S., Park H.M., Kim S., Paek N.-S. (2021). Antioxidant activity and short-chain fatty acid production of lactic acid bacteria isolated from Korean individuals and fermented foods. 3 Biotech.

[27] Sparavigna A. (2020). Role of the extracellular matrix in skin aging and dedicated treatment-state of the art. Plast. Aesthet. Res.

[28] Hachiya A., Sriwiriyanont P., Fujimura T., Ohuchi A., Kitahara T., Takema Y., Kitzmiller W.J., Visscher M.O., Tsuboi R., Boissy R.E. (2009). Mechanistic effects of long-term ultraviolet B irradiation induce epidermal and dermal changes in human skin xenografts. Am. J. Pathol..

[29] Yuksel Egrilmez M., Kocturk S., Aktan S., Oktay G., Resmi H., Simsek Keskin H., Guner Akdogan G., Ozkan S. (2022). Melatonin prevents UVB-induced skin photoaging by inhibiting oxidative damage and MMP expression through JNK/AP-1 signaling pathway in human dermal fibroblasts. Life.

[30] Kim J.-M., Chung K.-S., Yoon Y.-S., Jang S.-Y., Heo S.-W., Park G., Jang Y.-P., Ahn H.-S., Shin Y.-K., Lee S.-H. (2022). Dieckol isolated from *Eisenia bicyclis* ameliorates wrinkling and improves skin hydration via MAPK/AP-1 and TGF-β/Smad signaling pathways in UVB-irradiated hairless mice. Mar. Drugs.

[31] Kim M.-J., Woo S.W., Kim M.-S., Park J.-E., Hwang J.-K. (2014). Anti-photoaging effect of aaptamine in UVB-irradiated human dermal fibroblasts and epidermal keratinocytes. J. Asian Nat. Prod. Res..

[32] Quan T., Qin Z., Xia W., Shao Y., Voorhees J.J., Fisher G.J. (2009). Matrix-degrading metalloproteinases in photoaging. J. Investig. Dermatol. Symp. Proc..

[33] Noh E.-M., Lee G., Lim C.-H., Kwon K.B., Kim J.-M., Song H.-K., Yang H.J., Kim M.J., Kim M.-s., Lee Y.-R. (2022). Protective effects of Evodiae Fructus extract against ultraviolet-induced MMP-1 and MMP-3 expression in human dermal fibroblasts. J. Herb. Med..

[34] Brennan M., Bhatti H., Nerusu K.C., Bhagavathula N., Kang S., Fisher G.J., Varani J., Voorhees J.J. (2003). Matrix metalloproteinase-1 is the major collagenolytic enzyme responsible for collagen damage in UV-irradiated human skin. Photochem. Photobiol..

[35] Lee J.-J., Ng S.-C., Ni Y.-T., Liu J.-S., Chen C.-J., Padma V.V., Huang C.-Y., Kuo W.-W. (2021). Protective effects of galangin against H2O2/UVB-induced dermal fibroblast collagen degradation via hsa-microRNA-4535-mediated TGFβ/Smad signaling. Aging.

[36] Devos H., Zoidakis J., Roubelakis M.G., Latosinska A., Vlahou A. (2023). Reviewing the regulators of COL1A1. Int. J. Mol. Sci..

[37] Papakonstantinou E., Roth M., Karakiulakis G. (2012). Hyaluronic acid: A key molecule in skin aging. Derm. Endocrinol..

[38] Kang M.C., Yumnam S., Kim S.Y. (2018). Oral intake of collagen peptide attenuates ultraviolet B irradiation-induced skin dehydration in vivo by regulating hyaluronic acid synthesis. Int. J. Mol. Sci..

[39] Abe M., Masuda M., Mizukami Y., Inoue S., Mizutani Y. (2024). Epidermal keratinocytes regulate hyaluronan metabolism via extracellularly secreted hyaluronidase 1 and hyaluronan synthase 3. J. Biol. Chem..

