# Supplementary material for: Limosilactobacillus fermentum MG5368 and Lactiplantibacillus plantarum MG989 Regulates Skin Health in UVB-Induced HaCaT Cells and Hairless Mice Model
Source: Nutrients. 2024 Nov 27;16(23):4083. doi: 10.3390/nu16234083 (PMC11643855; doi:10.3390/nu16234083)
Supplement: Supplementary file 1 [file nutrients-16-04083-s001.zip › nutrients-3322627-supplementary.pdf]

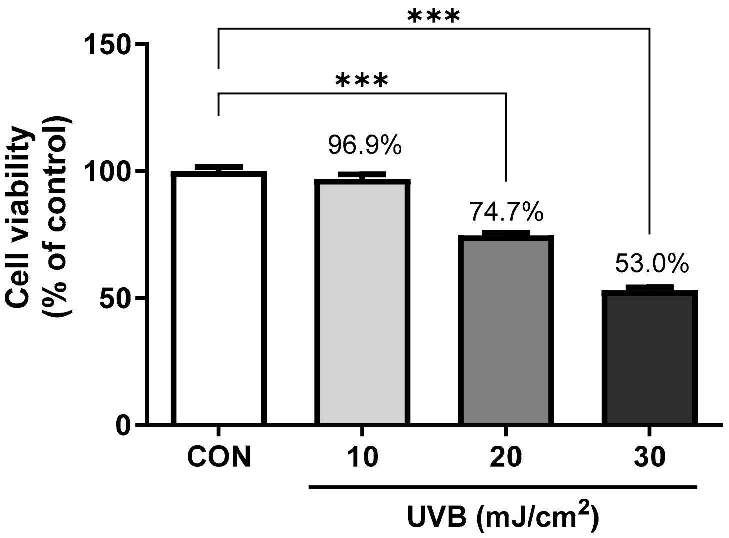

Figure S1. Cell viability of HaCaT keratinocyte cells after exposure to different doses of UVB (10, 20, and 30 mJ/cm^2). Values are expressed as the mean ± SEM (*n* = 3). ***$p < 0.001$, a significant difference compared to control.

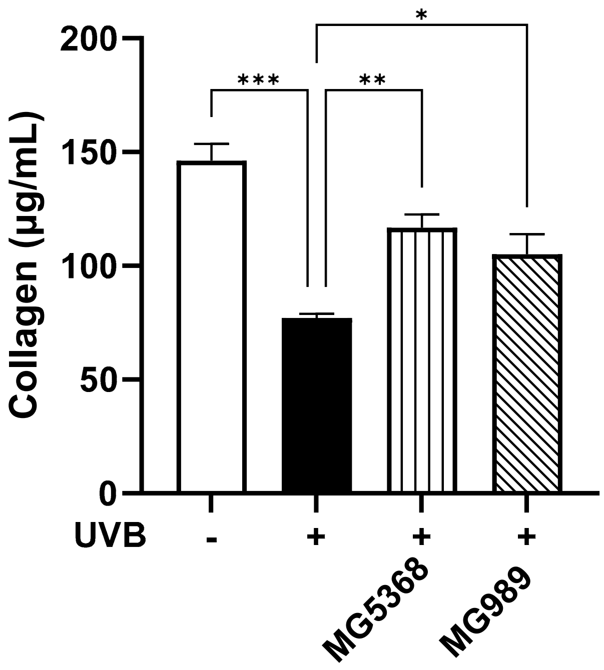

Figure S2. Effect on collagen content of CFS in UVB-exposed HaCaT keratinocytes. The cells were pre-treated with CFS (3%) for 24 h and then exposed UVB (20 mJ/cm^2) followed by additional treatment with CFS (3%) for 24 h. Values are expressed as the mean ± SEM (n = 3). * $p < 0.05$, ** $p < 0.01$ and *** $p < 0.001$, significant difference compared to UVB-exposed group.
